# Clinical manifestations of chronic pancreatitis in English cocker spaniels

**DOI:** 10.1111/jvim.17100

**Published:** 2024-05-09

**Authors:** M. Francisca Coddou, Barbara Blacklaws, Penny J. Watson

**Affiliations:** ^1^ Department of Veterinary Medicine University of Cambridge Cambridge UK

**Keywords:** anal sacculitis, canine, IgG4‐related disease, immune‐mediated disease, keratoconjunctivitis sicca, pancreas, proteinuria

## Abstract

**Background:**

Chronic pancreatitis (CP) is common in English cocker spaniels (ECS). It is histologically similar to IgG4‐related disease (IgG4‐RD) in humans and is characterized by duct destruction, interlobular fibrosis, and dense periductular and perivenous lymphocytic aggregates. However, the clinical manifestations of CP in ECS have not been previously described.

**Objectives:**

Characterize the clinical manifestations of CP in a group of ECS, including similarities and differences to IgG4‐RD in humans.

**Animals:**

One‐hundred four ECS with CP and 44 client owned control ECS without CP (both healthy and diseased controls).

**Methods:**

Affected dogs were divided into 2 groups according to the methods used to diagnose CP. Case records were searched for signalment, clinical, and clinicopathological findings, and evidence of keratoconjunctivitis sicca (KCS), proteinuria, other immune‐mediated diseases, and anal sacculitis.

**Results:**

Involvement of other organs was common. Affected ECS presented with a high frequency of KCS (n = 49), proteinuria (n = 47), anal gland disease (n = 36), atopy (n = 21), and other immune‐mediated diseases (n = 16). Those with parti‐color hair coats, particularly blue roan, had a strong association with CP, suggesting a link between coat color and autoimmune conditions in this breed.

**Conclusions and Clinical Importance:**

English cocker spaniels with CP show clinical similarities to humans with IgG4‐RD and common involvement of other organs. Clinicians should evaluate affected Cocker Spaniels for proteinuria, keratoconjunctivitis sicca, and other potential immune‐mediated diseases.

**Abbreviations**
CI: confidence interval
CP: chronic pancreatitis
cPLI: canine pancreatic lipase immunoreactivity
DGGR: 1,2‐*o*‐dilauryl‐rac‐glycero‐3‐glutaric acid‐(6′‐methylresorufin) ester
DM: diabetes mellitus
ECS: English cocker spaniels
EPI: exocrine pancreatic insufficiency
GN: glomerulonephritis
IBD: inflammatory bowel disease
IgG4‐RD: IgG4‐related disease
IMHA: immune‐mediated hemolytic anemia
IMPA: immune‐mediated polyarthritis
IMTP: immune‐mediated thrombocytopeniaa
KCS: keratoconjunctivitis sicca
OR: odds ratio
PCV: packed cell volume
STT: Schirmer tear test
UPC: urine protein: creatinine ratio

## 1 INTRODUCTION

English cocker spaniels suffer from a distinctive form of chronic pancreatitis (CP) histologically characterized by duct destruction, interlobular fibrosis, and dense periductular and perivenous lymphocytic aggregates with a predominance of IgG4‐positive plasma cells on immunohistochemistry in numerous organs including pancreas, kidney, salivary glands and orbital tissue.^1^, ^2^ Although not conclusively diagnostic of autoimmune disease, these distinctive features resemble those of autoimmune pancreatitis type 1 in humans, which is part of a steroid‐responsive, fibro‐inflammatory, multisystemic syndrome, currently known as IgG4‐related disease (IgG4‐RD).^3^ IgG4‐RD in humans can affect several organs although any organ can be involved, and a predominance of IgG4^+^ plasma cells is found histologically, with an IgG4^+^/total IgG^+^ plasma cell ratio >40%.^4^ Pancreas, lymph nodes, bile ducts, lacrimal glands, salivary glands, lungs, kidneys, aorta, meninges, and thyroid gland are the most commonly affected organs, and the majority of the patients have increased serum IgG4 concentrations.^5^ Moreover, an association between atopy and IgG4‐RD has been reported in human patients.^6^

Complete clinical findings in ECS with CP have not been reported previously. Involvement of kidney, salivary glands, and orbital tissue in affected ECS in a recent study of histological findings^2^ suggests that affected dogs may have clinical signs of glomerulonephritis (GN), dry eye, and dry mouth. Immune‐mediated cholangitis is also common in affected humans but was not prominent in histology of affected ECS. A possible association with anal sacculitis and anal sac carcinoma also has been suggested,^7^ and ECS with CP had an increased frequency of the same dog leukocyte antigen (DLA) type as ECS with anal sac carcinoma,^8^ as well as ECS with immune mediated hemolytic anemia (IMHA).^9^

Our aims were to describe the signalment and clinical findings in ECS with proven or suspected CP with a focus on other immune‐mediated diseases and anal sacculitis. Our hypothesis was that a high proportion of ECS with CP would have associated immune‐mediated diseases, particularly GN and dry eye. In addition, we hypothesized that many affected dogs also would have anal sac disease but would not have associated liver disease.

## 2 MATERIALS AND METHODS

### 2.1 Selection of cases and controls

English cocker spaniels affected by CP and client owned control ECS were prospectively recruited for this study between 2008 and 2019. Blood and serum samples were obtained from affected and control dogs and stored at −80°C until further analysis. Case material was obtained from dogs presented to the Queen's Veterinary School Hospital, University of Cambridge, and from private veterinary practices in the United Kingdom. Specifically, affected and control dogs were recruited with support from The Cocker Spaniel Club and The Kennel Club with the assistance of their breed health coordinators. Clinical records and surplus blood samples from clinical investigations were used with informed owner consent. Data regarding signalment and clinical status was collected from the clinical records. The study was approved by the Department of Veterinary Medicine, University of Cambridge Ethics Committee (CR153 and CR291).

#### 2.1.1 Cocker spaniels affected by CP

Chronic pancreatitis was diagnosed either by pancreatic histology or by increased lipase concentrations measured using a canine pancreatic lipase immunoreactivity (cPLI) test or a 1,2‐*o*‐dilauryl‐rac‐glycero‐3‐glutaric acid‐(6′‐methylresorufin) ester (DGGR) lipase assay or both, abnormal findings on pancreatic ultrasonography and presence of compatible clinical signs. All affected dogs had clinical signs consistent with CP (recurrent presentation with ≥2 of: vomiting, diarrhea, lethargy, anorexia, abdominal pain, and weight loss). Cases were divided into 2 groups because methods used to diagnose CP varied among dogs.

*Case group 1*: definite CP was diagnosed based on pancreatic histology and highly probable CP was diagnosed based on both an increased cPLI or DGGR lipase or both and pancreatic ultrasonographic findings consistent with pancreatitis.

*Case group 2*: likely CP was diagnosed based on increased cPLI in the absence of ultrasonographic findings, increased DGGR lipase, increased amylase or conventional lipase, and clinical signs consistent with CP.

Signalment details were recorded and case records were examined for the presence of any other disease conditions and with particular emphasis on diseases with reported histological associations with CP in ECS. The conditions were recorded as follows: keratoconjunctivitis sicca (KCS), diagnosed based on the Schirmer tear test (STT <15 mm/min) or a clinical diagnosis in the clinical records, GN (based on renal histology or suspected based on a urine protein: creatinine ratio [UPC] >1 on either a voided or cystocentesis urine sample) or a clinical diagnosis in the clinical records, anal sacculitis (history of recurrent expression of anal glands or infection), anal sac adenocarcinoma (histologically confirmed), atopic dermatitis, other immune‐mediated diseases, and diseases caused by end‐stage CP (eg, exocrine pancreatic insufficiency [EPI] and diabetes mellitus [DM]). Other concomitant diseases also were recorded.

#### 2.1.2 Control dogs

Age‐matched control dogs were included and were also divided into 2 groups. *Control group 1* consisted of healthy ECS. Surplus blood samples were obtained from ECS having pre‐anesthetic blood screening for routine procedures. *Control group 2* consisted of ECS affected by other conditions not related to CP and with no known immune‐mediated etiology. Surplus blood samples were obtained during routine clinical investigations in these dogs.

Inclusion criteria for control dogs consisted of no clinical evidence of CP, GN, KCS, anal sac disease or any other immune‐mediated diseases. Referring veterinarians were asked to perform STT on control dogs to rule out KCS and submit a blood and voided urine samples from each dog. Only dogs with normal DGGR lipase or Spec cPL or both and no previous clinical signs suggestive of CP, normal tear production, and UPC <0.4 were included as controls. Dogs that presented with normal or slightly increased DGGR lipase (<2*x* the upper limit of the reference interval) were used as controls only if they did not have any signs or history compatible with CP and the other diseases mentioned above. Exclusion criteria for control dogs consisted of corticosteroid or any other immunosuppressive treatment within the 6 months before obtaining the blood and urine samples.

#### 2.1.3 Clinical pathology

The DGGR lipase test, UPC ratio, packed cell volume (PCV), and platelet count were performed at Central Diagnostic Services, University of Cambridge, and were offered without charge for cases and controls. Many cases also had cPLI, serum biochemistry profiles, and CBCs performed during clinical investigations. Serum lipase activity was measured using a commercial DGGR lipase assay for dogs (Lipase assay DGGR; Randox) validated in house. The reference interval was 0 to 44 IU/L.^10^ However, a subsequent study from the same laboratory suggested that, using this reference interval, some control dogs may have mildly increased DGGR lipase but <3*x* the upper limit of the reference interval.^11^ Pancreatic lipase immunoreactivity was measured using the Spec cPL assay in most cases (Idexx laboratories, Wetherby, UK). Results >400 μg/L were considered positive for pancreatitis. Some cases were diagnosed with SNAP cPL (Idexx laboratories, Wetherby, UK).

Most cases had a complete urinalysis and negative sediment examination findings in addition to measurement of UPC. Most controls only had measurements of urine specific gravity and urine UPC.

#### 2.1.4 Other conditions and coat color

Atopy and other immune‐mediated diseases were diagnosed based on the clinical records of the attending veterinarian. Hypothyroidism was diagnosed based on concurrent low circulating thyroxine (T4) and high circulating thyroid‐stimulating hormone concentrations. Where available, coat color of both cases and controls was recorded. Coat colors were categorized according to the Kennel Club breed standards for the ECS: (https://www.thekennelclub.org.uk/breed-standards/gundog/spaniel-cocker/).

### 2.2 Statistical analysis

The ages of cases and controls were compared using the Mann‐Whitney *U* test. Odds ratios (OR) and 95% confidence intervals (CI) were calculated for disease association with coat color. Fisher's exact test was performed to determine if the OR was significant or not. The level of significance was set at *P* < .05. Statistical analysis was performed using SPSS software (IBM, version 22.0).

## 3 RESULTS

### 3.1 Clinical features in cases

**FIGURE 1:**
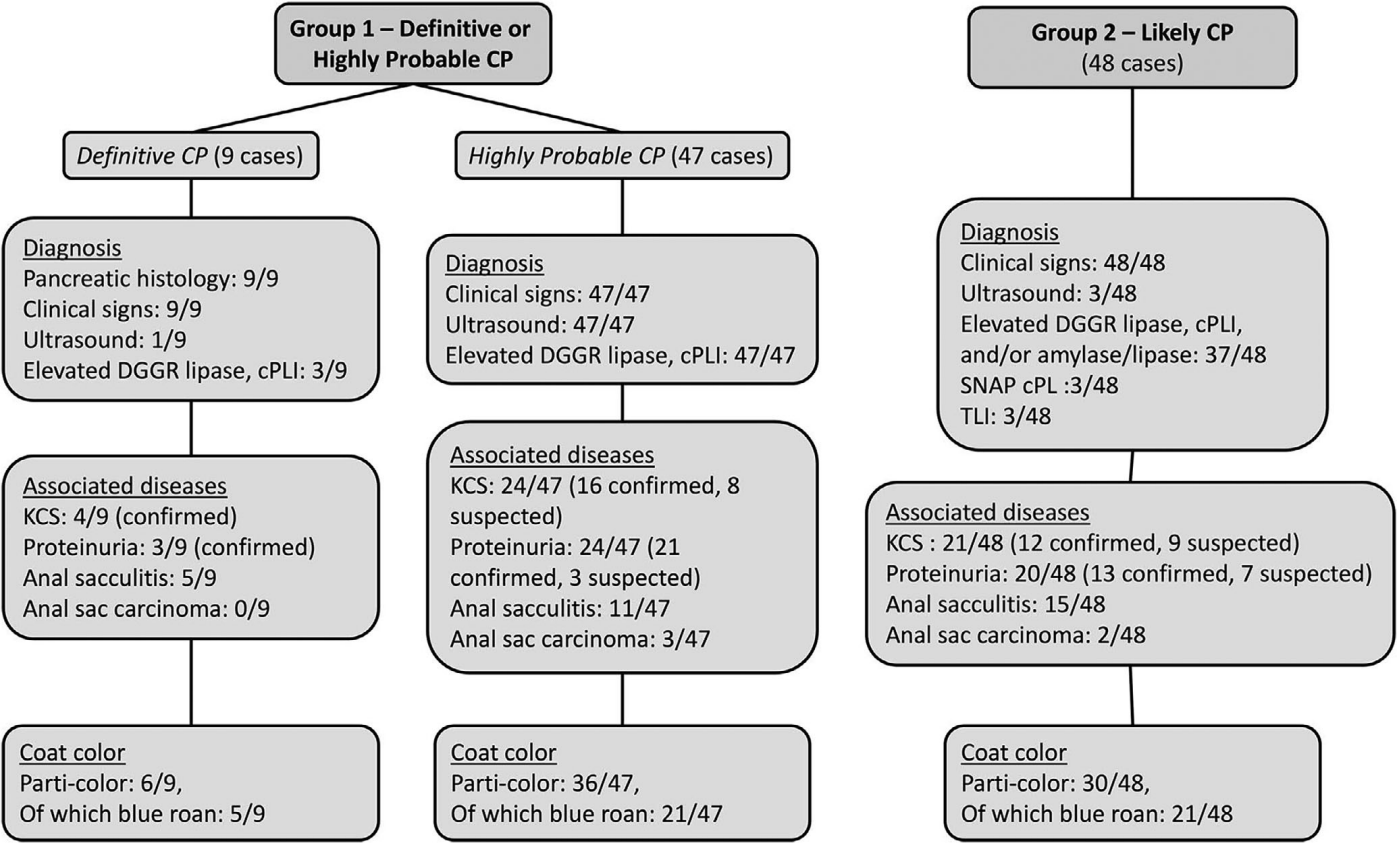
Flow chart showing criteria for recruitment of cases and controls and associated diseases and coat colors.

One‐hundred four ECS with CP were recruited. There were 51 females and 53 males. Median age was 9.1 years (range, 2.7‐16 years; Table 1). Group 1 included 56/104 (53.8%) CP cases (definite and confirmed on histology [n = 9] or highly probable [n = 47]) and group 2 (likely CP) included 48/104 (46.1%) cases. Figure 1 presents the groups and their clinical and diagnostic findings.

**TABLE 1:** Signalment and clinical details of cases and controls included in this study.

Category	Number of dogs	Age (years)		Sex					
		Median	Range	FE	FN	F	ME	MN	M
Cases	104	9.1	2.7‐16	2	46	3	7	38	8
Healthy control	15	10	7.0‐11.25	1	5	1	0	7	1
Diseased control	29	9.2	7.0‐13.3	0	10	0	4	13	2

Abbreviations: ECS, English cocker spaniel; F, female unknown neutering status; FE, female entire; FN, female neutered; M, male unknown neutering status; ME, male entire; MN, male neutered.

Lethargy, weight loss, inappetence, cranial abdominal pain, diarrhea, and intermittent vomiting were the most common clinical signs. Ultrasonographic changes in the pancreas compatible with CP were described in 47 cases. A heterogeneous mass‐like lesion in the pancreas was reported in 5/47 (10.6%) ECS that underwent ultrasonography.

Forty‐nine dogs were diagnosed with KCS (STT results available [n = 32]; history of KCS confirmed by referring veterinarian [n = 17]). Thirty‐eight of these dogs had bilateral KCS and the remaining 11 had unilateral KCS. Forty‐seven dogs presented with suspected or confirmed GN (suspected with UPC >1 [n = 34], with history of proteinuria [n = 10]; and confirmed on histology [n = 3]). The UPC median of the dogs with UPC performed was 4.1 (range, 1‐13.7). Thirty‐two dogs had UPC ≥2.

**FIGURE 2:**
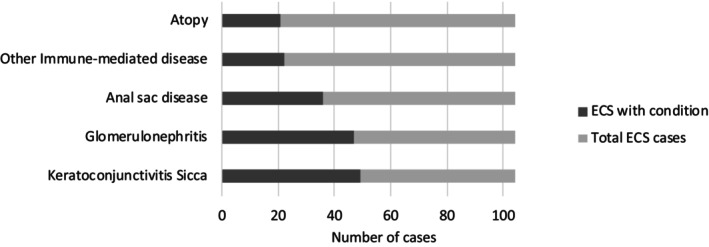
Proportion of ECS dogs affected by CP presenting with other concurrent conditions. [Correction added after first online publication on 31 May 2024. Figure 2 has been revised.].

Anal sac disease was identified in 36 dogs (anal sacculitis [n = 31]; anal sac adenocarcinoma [n = 5]). Twenty‐one dogs had a history of atopy. There was a history of other immune‐mediated conditions such as hypothyroidism (n = 6), immune‐mediated thrombocytopenia (IMTP; n = 4), IMHA (n = 4), immune‐mediated polyarthritis (IMPA; n = 2), inflammatory bowel disease (IBD; n = 4), and hypoadrenocorticism (n = 2) in 16 cases although no dogs were receiving immunosuppressive treatment for these conditions at the time of enrollment in the study. Table 2 summarizes the frequency of these conditions in affected ECS from groups 1 and 2. The proportion of cases presented with these conditions in the case study population is shown in Figure 2.

**TABLE 2:** Number of ECS dogs that presented with conditions associated with CP.

Condition		Number of cases group 1 (n = 56)	Number of cases group 2 (n = 48)	Total cases (n = 104)
Keratoconjunctivitis sicca	STT <15 mm/min	20	12	32
	History of dry eye	8	9	17
Glomerulonephritis	Biopsy/UPC >1	24	13	37
	History of proteinuria	3	7	10
Anal sac disease	Anal sacculitis	16	15	31
	Anal sac adenocarcinoma	3	2	5
Other immune‐mediated disease	Hypothyroidism	2	4	6
	Hypoadrenocorticism	2	0	2
	IMTP	1	3	4
	IMHA	0	4	4
	IMPA	0	2	2
	IBD	3	1	4
Atopy		8	13	21

Seventy‐five ECS with CP (72%) had involvement of other organs in addition to the pancreas, suggesting multiorgan chronic inflammatory disease (KCS, n = 49; GN, n = 47; anal sacculitis, n = 31). Thirty‐one ECS had 1 additional organ involved, 25 ECS had 2 organs involved, 16 ECS had 3 organs involved and 3 ECS had 4 organs involved in addition to the pancreas.

**FIGURE 3:**
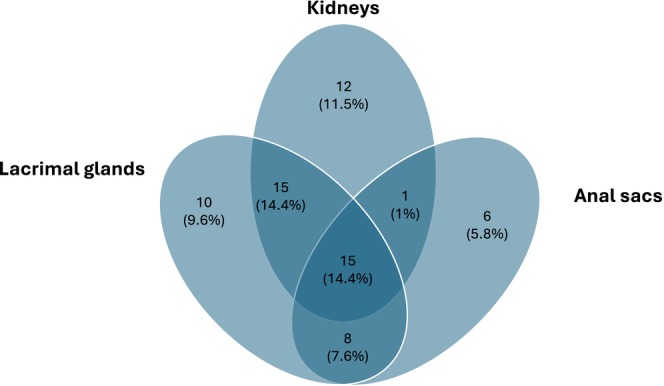
Number and percentage of ECSs affected by chronic pancreatitis with concurrent multiorgan pathology.

Figure 3 represents the number and percentage of cases having >1 organ affected by chronic inflammatory disease, showing an overlap in some affected organs.

Regarding diseases that may be caused by end‐stage CP, concurrent DM was present in 7/104 (6.7%) dogs and EPI was present in 11/104 (10.6%). Only 1 dog had both DM and EPI.

Regarding other diseases, 4/104 (3.8%) dogs had chronic hepatitis, 3/104 (2.9%) had intervertebral disc disease, 3/104 (2.9%) had idiopathic epilepsy, 3/104 (2.9%) had chronic bronchitis, 2/104 (1.9%) had osteoarthritis, 2/104 (1.9%) dogs had mammary masses, 2/104 (1.9%) dogs were cryptorchid, and 2/104 (1.9%) had enlarged prostate glands.

**FIGURE 4:**
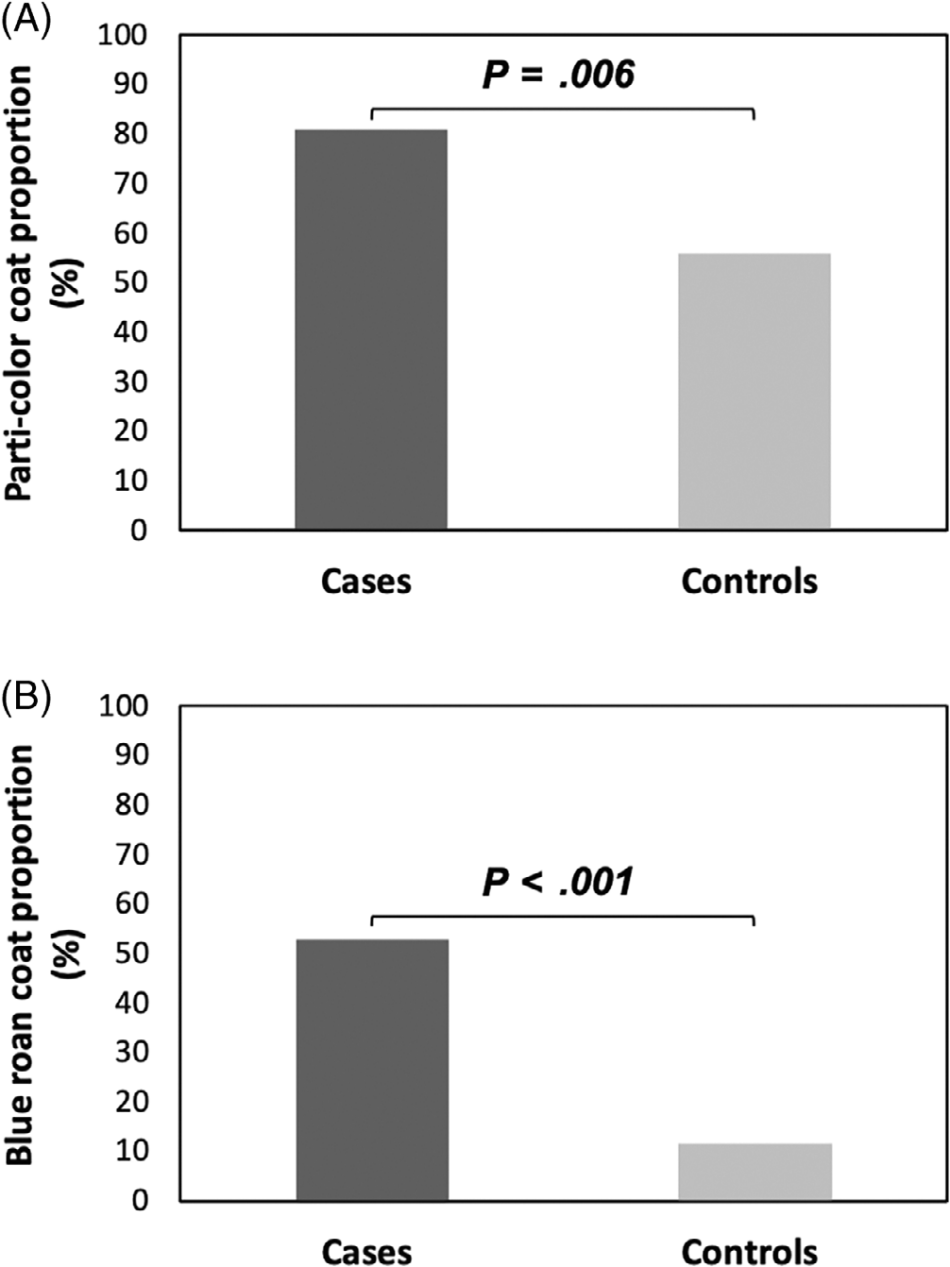
Comparison of proportions of different coat colors between cases and controls. (A) Parti‐color and (B) blue roan coat proportions in cases (dark gray) and controls (light gray). *P* values indicate statistical significance of differences.

Information regarding coat color was provided for 89/104 (85.6%) affected dogs. Of those dogs with coat color recorded, 72 (80.9%) had parti‐color coats (blue roan, n = 47 [52.8%]; black and white, n = 10 [11.2%]; tricolor, n = 5 [5.6%]; liver and white, n = 4 [4.5%]; orange roan, n = 5 [5.6%]; liver roan, n = 1 [1.1%]) and 17 (19.1%) had solid colors (golden, n = 8 [9.0%]; black, n = 4 [4.5%]; liver, n = 3 [3.4%]; red, n = 2 [2.2%]; Figure 4).

### 3.2 Clinical features in control dogs

Forty‐four ECS that fulfilled the strict inclusion criteria were recruited as controls. Fifteen dogs belonged to control group 1 (healthy ECS), and 29 belonged to control group 2 (diseased control group). Group 1 consisted of 7 females and 8 males and group 2 of 10 females and 19 males. Median age was 10.0 years for group 1 (range, 7.0‐11.3 years) and 9.2 years for group 2 (range, 7.0‐13.3 years; Table S1).

No significant difference was found in age between CP cases and controls (median age, 9.1 years, range 2.7‐16.0 years vs. 9.7 years; range 7.0‐13.3 years: Mann‐Whitney *U* test, *P* = .30).

Regarding diagnosis for control group 2, neurologic (10/29) and oncologic (9/29) cases were overrepresented in this study population. Seven dogs from control group 1 had slightly increased DGGR lipase (<2*x* above the upper limit of the laboratory reference interval) but did not have other signs of pancreatitis. Eight dogs from group 2 had slightly increased DGGR lipase (<2*x* the upper limit of the reference interval) with no other signs of pancreatitis. Five of them had intervertebral disc disease.

Information regarding coat color was obtained for 14/15 ECS from control group 1. Seven dogs had solid color coat (golden, [n = 3]; black, [n = 2]; liver, [n = 2]) and 7 had parti‐color coats (orange roan, [n = 2]; blue roan, [n = 2]; lemon roan, [n = 1]; lemon and white, [n = 1]; liver and white, [n = 1]).

Information about coat color in control group 2 was available for 20/29 dogs. Most of them had parti‐color coats (blue roan, [n = 2]; black and white, [n = 2]; orange roan, [n = 2]; liver and white, [n = 2]; tricolor, [n = 2]; lemon and white, [n = 1]; liver roan, [n = 1]) and 8 dogs had solid color coats (black, [n = 4]; golden, [n = 3]; and red [n = 1]).

Parti‐color coats had a significantly higher frequency in cases than in control dogs (80.9% vs 55.9%, *P* = .01). Blue roan parti‐color coat was overrepresented in cases compared with controls (52.8% vs 11.8%), indicating association of blue roan coat color with CP in ECS (odds ratio [OR], 8.39; 95% confidence interval [CI], 2.73‐25.80; *P* < .001; Figure 4).

Signalment and clinical details of case and control dogs can be found in Tables S1–S3.

## 4 DISCUSSION

We describe for the first time a large number of ECSs with CP compared with an age‐matched control group of the same breed. Dogs with other immune‐mediated diseases were excluded from the control group because surplus blood samples from some of these dogs were used for other studies of immunologic disease. In addition, exclusion criteria included immunosuppressive treatment in dogs in the control group because such treatment could mask relevant clinical signs. Affected dogs were significantly more likely to have parti‐colored coats compared with unaffected dogs and showed some similarities with IgG4‐related disease humans in that many dogs had concurrent potentially immune‐mediated diseases. However, unlike humans, very few affected dogs (2.9%) had concurrent liver disease. A high frequency of KCS was found in the ECS with CP in our study (47.1%), whereas other studies have shown a prevalence of KCS of 4% in the general dog population.^12^ Comparisons could not be made with the control population in our study because absence of KCS was an inclusion criterion for the control groups. In humans, orbital tissue frequently is involved in the course of IgG4‐RD. These patients often present with dry eye, which may be associated with lacrimal gland or orbital nerve involvement or both.^13^ The high frequency of KCS observed in our study suggests a similar link between CP and KCS in ECS. Therefore, it is relevant to evaluate tear production by the use of STT and determine the presence of dry eye in affected ECS. However, the prevalence of KCS in the ECS breed is unknown. Both CP and KCS could be common in ECS without necessarily having a shared pathogenesis.

In humans with IgG4‐RD, xerostomia (dry mouth resulting from decreased or absent saliva production) is present in 30% of patients. Histologic involvement of salivary glands (IgG4‐related sialadenitis) is observed in 27% to 53% of human patients with IgG4‐RD and submandibular glands are more frequently affected, but parotid, sublingual, and labial salivary glands also can be involved.^14^ We described infiltration of IgG4 positive plasma cells in the salivary glands of ECS in a previous histologic study.^2^ Difficulties in clinical evaluation and inconsistent reporting in cases and controls precluded evaluation of xerostomia in our study. Xerostomia is rarely evaluated in dogs but does occur,^15^ and it is a known cause of persistent dental disease in humans.^16^ Repeated dental treatments might be a surrogate marker for suspected xerostomia in dogs, but it would be difficult to determine the actual cause of persistent dental disease in dogs in a retrospective study. In humans, salivary flow is measured by stimulating saliva production by having the patient chew a piece of wax for 5 minutes and then expectorating. Such a procedure would not be easily applied to dogs. Further investigation would require a reliable method to measure salivary production in dogs. Assessing the size and firmness of salivary glands on clinical examination may be valuable in future studies of ECSs with CP.

In addition, proteinuria with suspected GN was common in ECS with CP (45.2%). Suspicion of GN in most dogs was based on a UPC >1 (32.7%) or by a history of proteinuria when a urine sample was not available (9.6%) and was confirmed on histology in only a few dogs (2.9%). In a manner similar to CP, GN be diagnosed on histopathology but renal biopsies are uncommonly performed in either humans or dogs because biopsy is a relatively invasive procedure. Proteinuria therefore was used as a surrogate marker of potential GN. Persistent proteinuria is a clinicopathological sign of GN, and a UPC >1 was used as a marker of possible GN in our study. A relatively low cut‐off for abnormal UPC was used so as not to miss any potential cases of GN. A UPC >1 is more than the borderline proteinuria as defined by the International Renal Interest Society (http://www.iris-kidney.com/pdf/IRIS-DOG-Treatment_Recommendations_2023.pdf). Ideally, repeat urine samples would have been taken to confirm abnormal UPC, as recommended by the American College of Veterinary Internal Medicine consensus statement on the diagnosis of glomerular disease in dogs,^17^ but doing so was not possible in our study because only 1 urine sample was submitted from each dog. Increased UPC is not specific for GN and could occur as a result of amyloidosis, hypertension, urinary tract infections, inflammation associated with ongoing CP or other disorders. Therefore suspicion of GN is only presumptive without a biopsy. Also several dogs did not have a UPC performed and their medical records simply mentioned the existence of proteinuria. Proteinuria alone is difficult to interpret because it varies with urine concentration and among different samples.^17^, ^18^ Sample collection and analysis methods differed between cases and controls. Most cases had complete urinalysis and sediment examination to rule out urinary tract inflammation whereas most controls did not, and most control samples were collected by voiding whereas many cases had cystocentesis performed. It is possible that some control dogs had unidentified active sediment which would have increased the UPC. Nonetheless, the UPC was significantly higher in the cases than controls.

In our study 29.8% of the ECS with CP had a history of anal sacculitis, whereas other studies have shown a prevalence of 12.5% for anal sacculitis in the general dog population.^19^ A previous unpublished histological study of anal sac tissue from ECS with anal sacculitis showed histopathological findings morphologically similar to IgG4‐RD in humans, with lymphocytic and plasmacytic infiltration, fibrosis, and increased numbers of IgG4^+^ plasma cells.^20^ This finding suggests that anal sacculitis in ECS could be an IgG4‐RD sequela. More studies are needed to define the pathophysiology of anal sacculitis in ECS compared with other breeds, including defining lymphocyte subsets and numbers on histology and response to immunosuppressive drugs.

Involvement of other organs (eg, kidney, lacrimal glands, anal sacs) in addition to the pancreas was seen in 72% of ECS with CP, suggesting a multisystemic chronic inflammatory disease, similar to IgG4‐RD. However, the possibility of co‐occurrence of independent disease entities should be considered rather than multiorgan disease. Also, the extent of involvement of other organs may have been underestimated because of a lack of relevant imaging studies or biopsy specimens.

Atopy and atopic dermatitis were present in 20.2% of our study population. Other studies have reported atopy and atopic dermatitis in approximately 10% of the general dog population.^21^ A potential link has been described between atopy and IgG4‐RD in humans, although it remains controversial. A study in the United Kingdom has reported a frequent clinical history of allergy (63%) and atopy (40%) in human patients with IgG4‐RD^6^ and similar findings have been reported in Japanese AIP patients^22^ but not in other studies.^23^ Concurrent immune‐mediated conditions such as hypothyroidism, IMTP, IMHA, IMPA, IBD, and hypoadrenocorticism were seen in 16 (15.4%) ECS with CP, supporting a predisposition to immune‐mediated disease, which is already recognized in the veterinary literature. Dogs affected by atopic dermatitis, IMTP or IMHA are frequently young to middle‐aged.^24^, ^25^ Dogs with CP frequently are older. In our study, the median age of affected ECS was 9.1 years whereas the mean age of dogs with IMHA in another study was 6.6 ± 2.9 years^25^ and most dogs with atopy present between 6 months and 3 years of age.^24^ These findings could suggest an effect of immunological aging on the type of disease. A previous genetic study showed an increased frequency of a single DLA haplotype in ECS with CP.^7^ This haplotype also is more frequent in ECS with IMHA,^9^ suggesting that CP may be part of a multiorgan immune‐mediated disease in ECS in which this DLA haplotype may play an important role.

Chronic pancreatitis causes permanent destruction of pancreatic tissue and progressive loss of exocrine and endocrine function, which eventually can progress to EPI or DM or both.^26^ These diseases reportedly develop only if 80% to 90% of functional pancreatic mass is lost, because the pancreas has extensive functional reserve capacity. Thus, many dogs will not reach end stage disease during their lives. A complex cause and effect relationship exists between pancreatitis and DM in dogs. A recent study in primary‐care clinics in the UK indicated that pancreatitis is associated with the risk of dogs developing DM. The study reported an apparent annual prevalence of DM of 0.26% and an annual incidence risk of 0.09% in dogs ≥3 years of age. The same study showed an increased hazard of death after diagnosis of DM in cocker spaniels compared with crossbreeds.^27^ No data currently is available on the prevalence of EPI in dogs with CP. In our study, 6.7% and 10.6% of ECS with CP had concurrent DM and EPI, respectively. These high frequencies emphasis the importance of actively evaluating their presence in ECS with CP.

The inflammatory lesions in IgG4‐related pancreatic disease in humans frequently cause diffuse enlargement of the organ or, in some cases, a benign pancreatic mass, making differentiation from pancreatic cancer difficult.^28^ In our study, only 5 affected dogs had mass‐like pancreatic lesions identified on ultrasonography. This pancreatic feature appears to be caused by fibrosis and inflammation.

Surprisingly, a strong association between CP and dogs with parti‐color coats was observed in our study (80.9% of the affected ECSs were parti‐color). In particular, the blue roan parti‐color coat was overrepresented in cases compared with controls (52.8% vs 11.8%), representing a significant association with CP in ECS (OR, 8.39; 95% CI, 2.73‐25.80; *P* < .001). This observation suggests a candidate gene in linkage disequilibrium with the gene that determines parti‐color coat. It has been demonstrated that pigment type switching in dogs is controlled by a gene that has been called the K locus, which is located on dog chromosome 16.^29^ This gene could be an interesting candidate gene to be further investigated in future genetic studies.

Fifteen dogs in the control population (7 from control group 1 and 8 from control group 2) had slightly increased serum DGGR activity. This increase was above the upper limit of the laboratory reference interval, but <3*x* the upper limit, which is the value often used clinically to signify a clinically relevant increase.^30^ This slight increase in the control dogs may be explained by hydrolysis of other lipases such as lipoprotein lipase or gastric lipase detected by the DGGR assay or could reflect a laboratory reference interval that is too narrow (which has been previously suggested^11^), normal biological variability, or the presence of pancreatic lesions that were not identified histologically because our control group did not have histology performed.^11^

Our study had limitations inherent in its initial design and control selection. Control dogs were included if they did not have evidence of CP but also if they did not have evidence of GN, KCS, anal sac disease or any other immune‐mediated diseases. Therefore, it was not possible to compare the prevalence of these diseases between groups. Future work including a control group without CP but also without exclusion of the conditions mentioned above would be needed to determine if these conditions are part of a multiorgan disease or if they are coexisting independent disease entities. Another limitation was the use of surrogate markers and not histopathology for the diagnosis of CP and GN. There was potential for bias in selection of both cases and controls such that they may not be representative of all ECS. However, involvement of the breed society in recruitment resulted in inclusion of dogs from multiple veterinary practices, which should minimize selection bias.

In conclusion, we characterized the clinical manifestations of CP in ECS and suggest potential clinical similarities to IgG4‐RD in humans, including high frequency of KCS, proteinuria, atopy and other concurrent immune‐mediated diseases that represent part of a multiorgan inflammatory syndrome. Anal sacculitis is also common in affected dogs. Especially interesting is the predisposition of parti‐color ECS, in particular blue roans, to CP. Future work is indicated, involving follow‐up of affected dogs to obtain current clinical records, development of a serum IgG4 test specific for dogs, and studies of the effects of immunosuppression in affected dogs to improve understanding of this disease and its manifestations.

## CONFLICT OF INTEREST DECLARATION

Authors declare no conflict of interest.

## OFF‐LABEL ANTIMICROBIAL DECLARATION

Authors declare no off‐label use of antimicrobials.

## INSTITUTIONAL ANIMAL CARE AND USE COMMITTEE (IACUC) OR OTHER APPROVAL DECLARATION

Approved by Department of Veterinary Medicine, University of Cambridge Ethics Committee (CR153 and CR291).

## HUMAN ETHICS APPROVAL DECLARATION

Authors declare human ethics approval was not needed for this study.

## Supporting information


**Table S1.** Signalment and clinical details of the 104 ECS cases affected by chronic pancreatitis included in this study.
**Table S2.** Signalment and clinical details of the 15 healthy control ECSs (control group 1) included in this study.
**Table S3.** Signalment and clinical details of the 29 control ECSs affected by other conditions unrelated to chronic pancreatitis and with no immune‐mediated etiology (control group 2) included in this study.

## References

[1] Watson PJ , Roulois A , Scase T , Holloway A , Herrtage ME . Characterization of chronic pancreatitis in English cocker spaniels. J Vet Intern Med. 2011;25:797‐804. 21689157 10.1111/j.1939-1676.2011.0744.x

[2] Coddou MF , Constantino‐Casas F , Scase T , Day MJ , Blacklaws B , Watson PJ . Chronic inflammatory disease in the pancreas, kidney and salivary glands of English cocker spaniels and dogs of other breeds shows similar histological features to human IgG4‐related disease. J Comp Pathol. 2020;177:18‐33. doi:10.1016/j.jcpa.2020.03.008 32505237

[3] Perugino CA , Stone JH . IgG4‐related disease: an update on pathophysiology and implications for clinical care. Nat Rev Rheumatol. 2020;16(12):702‐714. doi:10.1038/s41584-020-0500-7 32939060

[4] Culver EL , Bateman AC . General principles of IgG4‐related disease. Diagnostic Histopathol. 2013;19(4):111‐118. doi:10.1016/j.mpdhp.2013.01.003

[5] Ardila‐Suarez O , Abril A , Gómez‐Puerta JA . IgG4‐related disease: a concise review of the current literature. Reumatol Clin. 2017;13(3):160‐166. 27329319 10.1016/j.reuma.2016.05.009

[6] Culver EL , Sadler R , Bateman AC , et al. Increases in IgE, eosinophils, and mast cells can be used in diagnosis and to predict relapse of IgG4‐related disease. Clin Gastroenterol Hepatol. 2017;15(9):1444‐1452. 28223204 10.1016/j.cgh.2017.02.007 PMC5592233

[7] Bazelle J , Aguirre‐Hernandez J , Watson P , Kennedy L . Association between chronic pancreatitis and dog leukocyte antigen haplotypes in the English cocker spaniel. In: Proceedings of the ACVIM Forum, Seattle and Published in the J Vet Intern Med; 2013.

[8] Aguirre‐Hernández J , Polton G , Kennedy LJ , Sargan DR . Association between anal sac gland carcinoma and dog leukocyte antigen‐DQB1 in the English cocker spaniel. Tissue Antigens. 2010;76(6):476‐481. 20727114 10.1111/j.1399-0039.2010.01554.x

[9] Kennedy LJ , Barnes A , Ollier WER , Day MJ . Association of a common dog leucocyte antigen class II haplotype with canine primary immune‐mediated haemolytic anaemia. Tissue Antigens. 2006;68(6):502‐508. 17176441 10.1111/j.1399-0039.2006.00715.x

[10] Serrano G , Paepe D , Williams T , Watson P . Increased canine pancreatic lipase immunoreactivity (cPLI) and 1,2‐o‐dilauryl‐rac‐glycero‐3‐glutaric acid‐(6′‐methylresorufin) ester (DGGR) lipase in dogs with evidence of portal hypertension and normal pancreatic histology: a pilot study. J Vet Diagn Investig. 2021;33(3):548‐553. 33797297 10.1177/10406387211003987 PMC8120087

[11] Thomson JM , Williams TL . Retrospective analysis of association between hepatopathy and serum DGGR lipase activity in dogs: a pilot study. J Vet Diagn Investig. 2022;34(5):854‐858. 35762109 10.1177/10406387221106401 PMC9446298

[12] Williams DL . Immunopathogenesis of keratoconjunctivitis sicca in the dog. Vet Clin North Am Small Anim Pract. 2008;38(2):251‐268. 18299006 10.1016/j.cvsm.2007.12.002

[13] Kocabeyoglu S , Karadag O , Mocan MC , Erden A , Irkec M . Orbital involvement and ocular surface changes in IgG4‐related systemic disease. Cornea. 2016;35(11):1449‐1453. 27467041 10.1097/ICO.0000000000000965

[14] Puxeddu I , Capecchi R , Carta F , Tavoni AG , Migliorini P , Puxeddu R . Salivary gland pathology in IgG4‐related disease: a comprehensive review. J Immunol Res. 2018;2018:1‐7. 10.1155/2018/6936727 PMC5901485 29805984

[15] Nabeta R , Kambe N , Nakagawa Y , et al. Sjögren's‐like syndrome in a dog. J Vet Med Sci. 2019;81(6):886‐889. 31092740 10.1292/jvms.18-0387 PMC6612494

[16] Cassolato SF , Turnbull RS . Xerostomia: clinical aspects and treatment. Gerodontology. 2003;20(2):64‐77. 14697016 10.1111/j.1741-2358.2003.00064.x

[17] Littman MP , Daminet S , Grauer GF , Lees GE , van Dongen AM . Consensus recommendations for the diagnostic investigation of dogs with suspected glomerular disease. J Vet Intern Med. 2013;27:S19‐S26. 24635376 10.1111/jvim.12223

[18] Meindl AG , Lourenço BN , Coleman AE , Creevy KE . Relationships among urinary protein‐to‐creatinine ratio, urine specific gravity, and bacteriuria in canine urine samples. J Vet Intern Med. 2019;33(1):192‐199. 30506746 10.1111/jvim.15377 PMC6335512

[19] Halnan CRE . The frequency of occurrence of anal sacculitis in the dog. Small Anim Pract. 1976;17:537‐541. 10.1111/j.1748-5827.1976.tb06997.x 966733

[20] Coddou MF . An Investigation of Polysystemic Immune‐Mediated Disease in the English Cocker Spaniel [MPhil Thesis]. University of Cambridge. 2015.

[21] Scott DW , Miller WH , Griffin CE . Muller & Kirk's Small Animal Dermatology. 6th ed. Philadelphia: W.B. Saunders; 2001:574‐601.

[22] Kamisawa T , Anjiki H , Egawa N , Kubota N . Allergic manifestations in autoimmune pancreatitis. Eur J Gastroenterol Hepatol. 2009;21(10):1136‐1139. 19757521 10.1097/meg.0b013e3283297417

[23] Della TE , Mattoo H , Mahajan VS , Carruthers M , Pillai S , Stone JH . Prevalence of atopy, eosinophilia, and IgE elevation in IgG4‐related disease. 2014;69(6):269‐272. 10.1111/all.12320 PMC4638385 24266692

[24] Gedon NKY , Mueller RS . Atopic dermatitis in cats and dogs: a difficult disease for animals and owners. Clin Transl Allergy. 2018;8(1):1‐12. doi:10.1186/s13601-018-0228-5 30323921 PMC6172809

[25] Weinkle TK , Center SA , Randolph JF , Warner KL , Barr SC , Erb HN . Evaluation of prognostic factors, survival rates, and treatment protocols for immune‐mediated hemolytic anemia in dogs: 151 cases (1993‐2002). J Am Vet Med Assoc. 2005;226(11):1869‐1880. 15934255 10.2460/javma.2005.226.1869

[26] Watson PJ . Exocrine pancreatic insufficiency as an end stage of pancreatitis in four dogs. J Small Anim Pract. 2003;44:306‐312. 12866928 10.1111/j.1748-5827.2003.tb00159.x

[27] Heeley AM , O'Neill DG , Davison LJ , Church DB , Corless EK , Brodbelt DC . Diabetes mellitus in dogs attending UK primary‐care practices: frequency, risk factors and survival. Canine Med Genet. 2020;7(1):1‐19.32835227

[28] Stone JH , Zen Y , Deshpande V . IgG4‐related disease. 2012;539–551. 10.1056/NEJMra1104650 22316447

[29] Kerns JA , Cargill EJ , Clark LA , et al. Linkage and segregation analysis of black and brindle coat color in domestic dogs. Genetics. 2007;176(3):1679‐1689. 17483404 10.1534/genetics.107.074237 PMC1931550

[30] Prümmer JK , Howard J , Grandt LM , Obrador de Aguilar R , Meneses F , Peters LM . Hyperlipasemia in critically ill dogs with and without acute pancreatitis: prevalence, underlying diseases, predictors, and outcome. J Vet Intern Med. 2020;34(6):2319‐2329. 32945588 10.1111/jvim.15902 PMC7694860

