# Supplementary material for: Clinical manifestations of chronic pancreatitis in English cocker spaniels
Source: J Vet Intern Med. 2024 May 9;38(4):2129–37. doi: 10.1111/jvim.17100 (PMC11256184; doi:10.1111/jvim.17100)
Supplement: Supplementary file 1 — Table S1. Signalment and clinical details of the 104 ECS cases affected by chronic pancreatitis included in this study. Table S2. Signalment and clinical details of the 15 healthy control ECSs (control group 1) included in this study. Table S3. Signalment and clinical details of the 29 control ECSs affected by other conditions unrelated to chronic pancreatitis and with no immune‐mediated etiology (control group 2) included in this study. [file JVIM-38-2129-s001.docx]

Table 1: Signalment and clinical details of the 104 ECS cases affected by chronic pancreatitis included in this study.

ECS, English cocker spaniel; F, female; FN, neutered female; M, male; MN, neutered male; CP, chronic pancreatitis; GN, glomerulonephritis; KCS, keratoconjunctivitis sicca; AS, anal sacculitis; ASAC, anal sac adenocarcinoma; EPI, exocrine pancreatic insufficiency; EHBO, extra hepatic biliary tract obstruction; DM, diabetes mellitus; IBD, inflammatory bowel disease; IMHA, immune mediated haemolytic anaemia; IMPA, immune mediated polyarthritis; IMTP, immune mediated thrombocytopenia; PLE, protein-losing enteropathy; NR, not recorded. * Working cocker spaniel.

Table 2: Signalment and clinical details of the 15 healthy control ECSs (control group 1) included in this study.

ECS, English cocker spaniel; F, female; FN, neutered female; M, male; MN, neutered male; NR, not recorded.

Table 3: Signalment and clinical details of the 29 control ECSs affected by other conditions unrelated to chronic pancreatitis and with no immune-mediated aetiology (control group 2) included in this study.

ECS, English cocker spaniel; F, female; FN, neutered female; M, male; MN, neutered male; DCM, dilated cardiomyopathy; MVD, mitral valve disease; USMI, urethral sphincter mechanism incompetence; IVDD, intervertebral disc disease; TCC, transitional cell carcinoma; NR, not recorded.
